# Regulation of Transcriptional Activity of Merkel Cell Polyomavirus Large T-Antigen by PKA-Mediated Phosphorylation

**DOI:** 10.3390/ijms24010895

**Published:** 2023-01-03

**Authors:** Mar Falquet, Carla Prezioso, Maria Ludvigsen, Jack-Ansgar Bruun, Sara Passerini, Baldur Sveinbjørnsson, Valeria Pietropaolo, Ugo Moens

**Affiliations:** 1 Molecular Inflammation Research Group, Department of Medical Biology, University of Tromsø-The Arctic University of Norway, 9037 Tromsø, Norway; 2 Microbiology of Chronic Neuro-Degenerative Pathologies, IRCSS San Raffaele, 00163 Rome, Italy; 3 Department of Public Health and Infectious Diseases, Sapienza University of Rome, 00185 Rome, Italy; 4 Department of Medical Biology, Proteomics Platform, University of Tromsø-The Arctic University of Norway, 9037 Tromsø, Norway; 5 Childhood Cancer Research Unit, Department of Women’s and Children’s Health, Karolinska Institute, 17177 Stockholm, Sweden

**Keywords:** Merkel cell carcinoma, Merkel cell polyomavirus, large T-antigen, PKA, phosphorylation, forskolin

## Abstract

Merkel cell polyomavirus (MCPyV) is the major cause of Merkel cell carcinoma (MCC), an aggressive skin cancer. MCPyV large T-antigen (LTag) and small T-antigen (sTag) are the main oncoproteins involved in MCPyV-induced MCC. A hallmark of MCPyV-positive MCC cells is the expression of a C-terminal truncated LTag. Protein kinase A (PKA) plays a fundamental role in a variety of biological processes, including transcription by phosphorylating and thereby regulating the activity of transcription factors. As MCPyV LTag has been shown to be phosphorylated and acts as a transcription factor for the viral early and late promoter, we investigated whether LTag can be phosphorylayted by PKA, and whether this affects the transcript activity of LTag. Using a phosphorylation prediction algorithm, serine 191, 203, and 265 were identified as putative phosphorylation sites for PKA. Mass spectrometry of in vitro PKA-phosphorylated peptides confirmed phosphorylation of S203 and S265, but not S191. Full-length LTag inhibited early and late promoter activity of MCPyV, whereas the truncated MKL2 LTag variant stimulated both promoters. Single non-phosphorylable, as well as phosphomimicking mutations did not alter the inhibitory effect of full-length LTag. However, the non-phosphorylable mutations abrogated transactivation of the MCPyV promoters by MKL2 LTag, whereas phosphomimicking substitutions restored the ability of MKL2 LTag to activate the promoters. Triple LTag and MKL2 LTag mutants had the same effect as the single mutants. Activation of the PKA signaling pathway did not enhance MCPyV promoter activity, nor did it affect LTag expression levels in MCPyV-positive Merkel cell carcinoma (MCC) cells. Our results show that phosphorylation of truncated LTag stimulates viral promoter activity, which may contribute to higher levels of the viral oncoproteins LTag and sTag. Interfering with PKA-induced LTag phosphorylation/activity may be a therapeutic strategy to treat MCPyV-positive MCC patients.

## 1. Introduction

Merkel cell carcinoma (MCC) is a highly aggressive cutaneous neuroendocrine cancer caused by either UV light exposure (approximately 20% of the cases) or by a human polyomavirus assigned Merkel cell polyomavirus (MCPyV). Characteristic features for MCPyV-positive MCCs are the integration of the viral genome and expression of a C-terminal truncated variant of one of the viral proteins, large T-antigen (LTag) [1,2]. LTag is necessary for viral DNA replication, by binding to repeated 5′-GRGGC-3′ motifs in the origin of replication, but also by its intrinsic helicase/ATPase activity and by recruiting cellular proteins involved in replication. Moreover, LTag controls the activity of the viral early and late promoter, which governs transcription of the early genes and late genes, respectively. The early genes encode LTag, small t-antigen (sTag), 57 kT, and ALTO, whereas the late genes encode the capsid proteins VP1 and VP2 [3,4,5]. LTag and sTag are oncoproteins that play a role in tumorigenesis and tumor progression [6,7,8,9].

**Figure 1:**
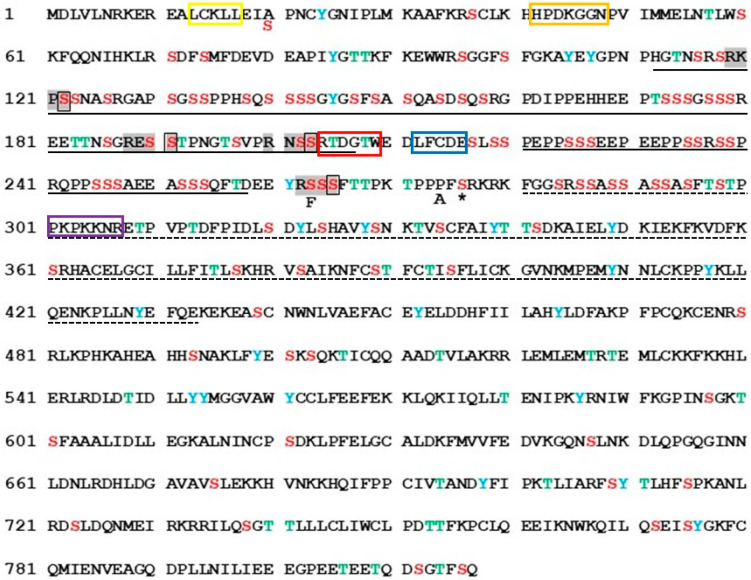
Amino acid sequence of MCPyV full-length large T-antigen (strainR17b; accession number NC_0102777). Putative phosphoacceptor serine (S), threonine (T), and tyrosine (Y) residues are highlighted in red, green, and blue, respectively. The sequence of the truncated large T-antigen variant MKL2 from a MCPyV-positive MCC tumor is identical except for S in position 20, phenylalanine (F) in position 263, alanine (A) in position 274, and stop (*) at residue 276. The PKA consensus motifs (RxxS) are highlighted in grey and the putative phosphoacceptor site is boxed. The functional CR1 domain (yellow), DnaJ domain (orange), hVam6p (red), pRb binding domain (blue), and NLS (purple) are boxed. The MCPyV large T-antigen unique regions MUR1 and MUR are underlined by a full line, and the origin binding domain is underlined by a dashed line. The C-terminal part of large T-antigen encompasses the ATPase/helicase activity.

MCPyV LTag is a817 amino acids long and has a statistical overrepresentation of the serine (S), threonine (T), and 24 tyrosine (Y) residues in the N-terminal domain (Figure 1). These residues form putative phosphoacceptor sites for protein kinases. Previous studies have shown that LTag can be phosphorylated at several residues, but which protein kinases are responsible for phosphorylation, and the biological consequence of phosphorylation are incompletely known (Supplementary Figure S1 and Supplementary Table S1). Mutations of some residues resulted in increased stability of LTag (S147A and S239A; [10,11]), whereas others partially inhibited the growth of MCPyV-positive cells (S219A; [12]). The T297A mutant displayed increased binding to the viral origin of replication and increased replication of viral DNA compared to wild-type LTag, whereas the opposite was observed for the T299A mutant [13]. S816 was shown to be phosphorylated by ataxia-telangiectasia mutated protein kinase and the S816A mutation partially reversed inhibition of C33A cell growth compared to wild-type LTag, and reduced apoptosis [10,14]. The S220A mutant inhibited growth of the MCPyV-positive MKL-1 MCC cell line and impaired binding to the retinoblastoma protein. Moreover, this mutant showed increased half-life and LTag-dependent viral DNA replication compared to wild-type LTag [10,11,12,15].

Phosphorylation of proteins is a major posttranslational modification, and it may regulate several functions of a protein such as its subcellular localization, activity, stability, and interaction with other partners [16]. Phosphorylation occurs on Ser, Thr, and Tyr residues by specific protein kinases. Many signaling pathways transmit, amplify, and convert signals through phosphorylation of a cascade of proteins, ultimately resulting in a cellular response [17,18]. Ser/Thr phosphorylation is typical for the mitogen-activated protein kinase (MAPK) pathways which consist of a cascade of the Ser/Thr kinases MAPK kinase kinase, MAPK kinase, MAPK, and MAPK-activated protein kinase (MAPKAPK) which phosphorylate and activate their downstream kinase to ultimately phosphorylate target proteins resulting in cellular responses [19]. Tyr phosphorylation is typical for receptor tyrosine kinases (RTKs) pathways. RTKs are receptors with intrinsic Tyr kinase activity. Upon ligand binding, RTKs form dimers and transphosphorylate the subunits at Tyr residues, further transmitting the signal into the cell to trigger a downstream effect [20]. One of the major intracellular signaling pathways is the cAMP-dependent protein kinase pathway or the protein kinase A (PKA) pathway. PKA is a tetrameric enzyme that consists of two regulatory subunits that bind cAMP, and two catalytic subunits with protein kinase activity. Binding of cAMP to the regulatory subunits results in the release of the catalytic subunits, which can then phosphorylate substrates at S or T, in the consensus motif RXXS/T [21]. One of the major substrates of PKA is the transcription factor CREB (cAMP response element binding protein) [22,23]. The cAMP-dependent protein kinase/CREB pathway has been demonstrated to activate the promoter of human polyomavirus BK [24], and mouse polyomavirus LTag can activate CREB binding sites [25]. MCPyV LTag has been shown to be phosphorylated, and acts as a transcription factor for the viral early and late promoter. As PKA can phosphorylate and modulate the activity of transcription factors, we investigated whether LTag can be phosphorylated by PKA, and explored whether this post-translational modification had an effect on the transcript activity of full-length LTag and a truncated LTag variant, expressed in the MKL2 MCC cell line. Briefly, our results show that in vitro phosphorylation of S203 and S265 by PKA and PKA mediated phosphorylation affected the transcriptional activity of the truncated MKL2 LTag variant, but not of full-length LTag.

## 2. Results

### 2.1. Identification of Putative PKA Phosphoacceptor Sites

Phosphorylation is one of the major post-translational modification mechanisms to regulate the behavior of a protein [26]. MCPyV LTag has been shown to be a phosphoprotein, but little is known about the protein kinases that mediate phosphorylation of LTag [12,13,14]. More than three hundred putative and proven cellular substrates of PKA have been described [27,28,29,30]. This prompted us to examine whether MCPyV LTag could be a PKA substrate. Using NetPhos 3.1 program to predict the PKA phosphorylation site [31], four putative PKA phosphoacceptor sites were identified (Figure 1): S122 (RKPS PKA motif), S191 (RESS PKA motif), S203 (RNSS PKA motif), and S265 (RSSS PKA motif). S191 and S203 are conserved in almost all MCPyV LTag sequences deposited in GenBank, whereas S265 is not present in truncated LTag version of MCPyV-positive MCCs (see Supplementary Table S2). Mass spectrometry analysis of ectopically expressed truncated LTag, from the MCC cell line WaGa, showed that phosphorylation of S265, and at least one amino acid within a peptide fragment containing S202, S203, and T205 was phosphorylated. No phosphorylation was detected for S122 and S191 [12]. We decided to investigate S191, S203, and S265 because they had a high prediction score (0.685, 0.671, and 0.578, respectively), are the acceptor residues in the PKA consensus RXXS/T, and are conserved in all available LTag sequences. S122 was not considered because it had a lower prediction score (0.486), was not found to be phosphorylated by mass spectrometry studies [12], and is not conserved in known MCPyV LTag sequences [32,33].

### 2.2. Mass Spectrometry of In Vitro Phosphorylated Peptides Suggest S203 and S265 as PKA Phosphoacceptor Residues

**Figure 2:**
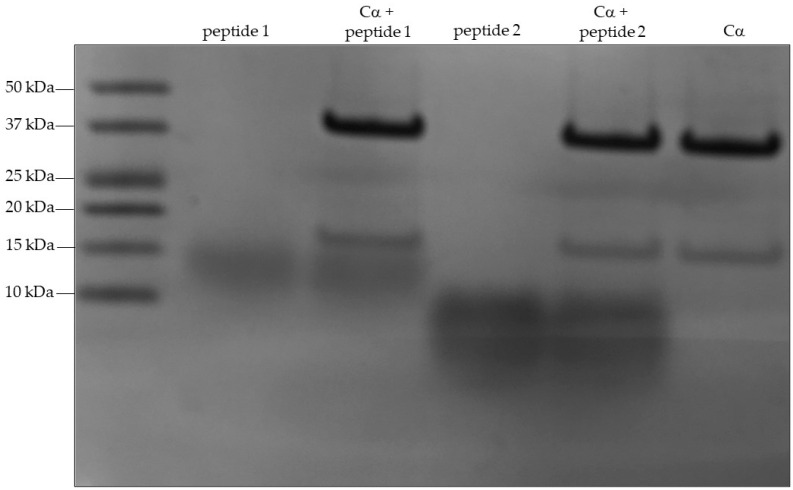
Coomassie staining of peptide 1, peptide 2, and the catalytic subunit of PKA. Five mg peptide was incubated with PKA for 30 min and thereafter run on an acryl amide gel. Proteins were stained with Coomassie blue. The molecule mass marker is shown (in kDa).

To test whether PKA could phosphorylate S191, S203, and S265, two peptides were synthesized. Peptide one encompassed S191 and S203, whereas peptide 2 contained S265 (see Section 4.6 for the peptide sequences). Before running an in vitro kinase assay on the peptides, we analyzed the peptides (2.5 μg) and the recombinant catalytic subunit of PKA (1 μg) on an acryl amide gel and visualized the peptides by Coomassie blue staining (Figure 2). A clear band of > 37 kDa is observed in the lanes with the recombinant catalytic subunit of PKA and corresponds well to the calculated molecular mass of 42.5 kDa [34]. The diffuse bands represent peptide 1 (33 amino acids) and 2 (21 residues), respectively. After in vitro analysis of kinase with the recombinant catalytic subunit of PKA, the peptides were subjected to mass spectrometry. The results showed that phosphorylation occurred on S202 and/or S203 and on S264 and/or S265. No phosphorylation was found on S191 (Supplementary Table S2 and Supplementary Figure S2).

### 2.3. Mutating S191, S203 and S265 Has No Significant Effect the Inhibitory Effect ofMCPyV Full-Length LTag on the Viral Early and Late Promoter

**Figure 3:**
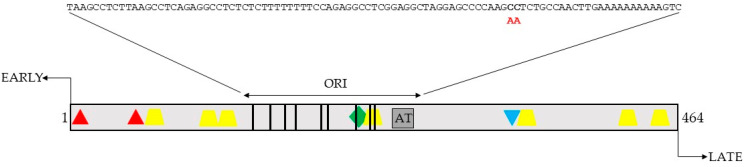
The NCCR of MCPyV with the origin of replication (ORI). The sequence of the ORI is shown and the CC into AA mutation, to generate mutE and mutL, is depicted in red. The early and late orientation of the NCCR is indicated. The vertical lines represent LTAg binding motifs (GRGGC). Putative binding sites for the transcription factors Sp1 (yellow trapezium), NF1 (green diamond), and STAT (blue triangle) are shown. The red triangles represent TATA boxes. AT is an AT-rich sequence.

Previous studies in other cell lines have shown that MCPyV LTag represses the MCPyV early and the late promoter [10,35]. We therefore tested the effect of LTag on the MCPyV early and late promoter activity, in HaCaT cells. HaCaT cells are a spontaneously immortalized human keratinocyte cell line derived from adult skin [36]. Because keratinocytes have been suggested to be a cell of origin for Merkel cell carcinoma, we decided to use this cell line (reviewed in [37,38]. The promoter sequences are part of the non-coding control region, which overlap with sequences required for viral DNA replication. Viral replication is stimulated by LTag. To avoid replication of the luciferase reporter plasmids containing either the early or the late promoter sequences in the presence of LTag, replication deficient origin was used, as previously described [10]. The luciferase reporter plasmids containing the replication deficient NCCR are referred to as mutE and mutL, respectively, and a schematic presentation of the NCCR with the mutation is shown in Figure 3.

**Figure 4:**
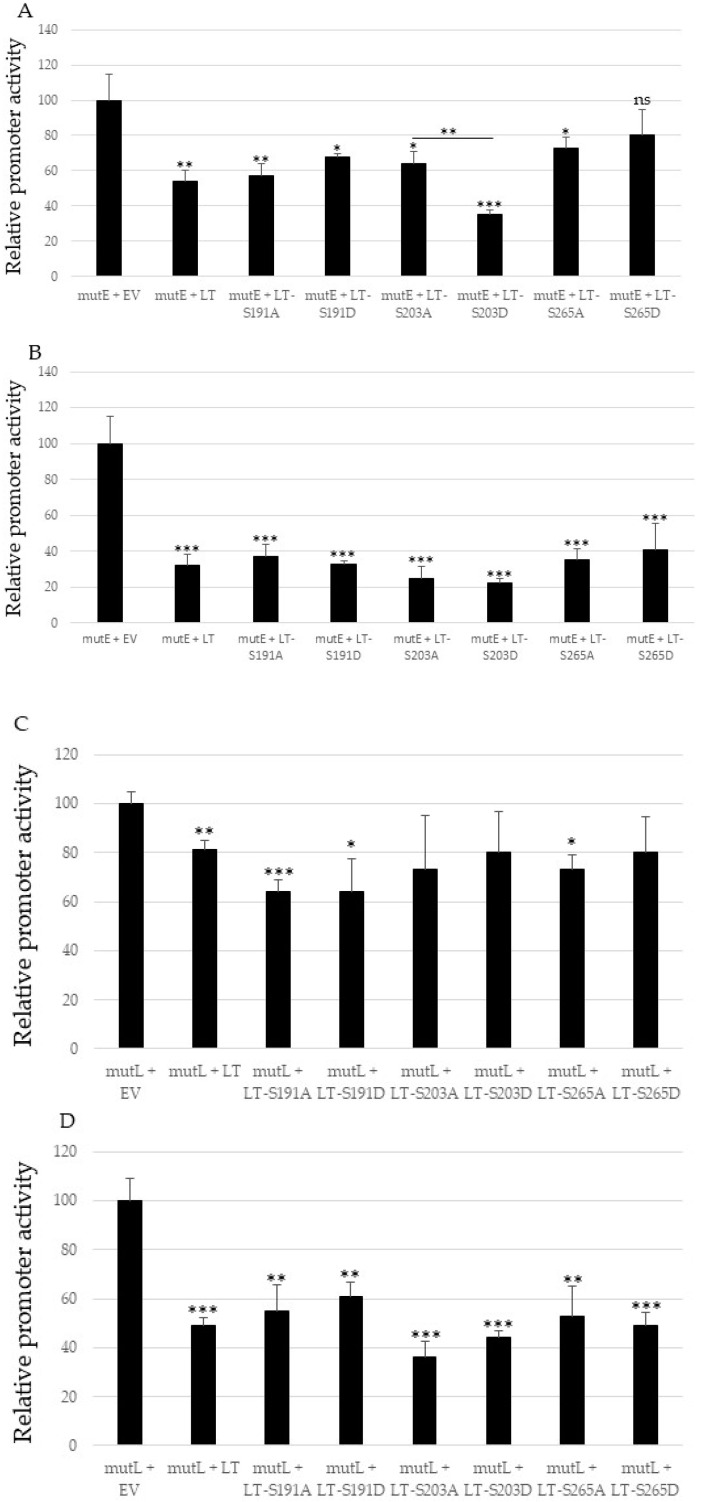
Full-length LTag and single mutants in the PKA phosphoacceptor sites inhibit MCPyV early and late promoter activity. HaCaT cells were co-transfected with 1 μg luciferase reporter plasmid containing the replication deficient early (**A**,**B**), or late promoter (**C**,**D**) and 100 ng (**A**,**C**), or 400 ng (**B**,**D**), empty vector (EV), or expression plasmid for full-length LTag, or the single non-phosphorylable (S replaced by A) or phosphomimicking (S substituted by D) mutants in S191, S203, and S265, respectively. Each bar represents the average of three independent parallels with standard deviation. Statistically significant differences with empty expression vector are indicated (* *p* < 0.05; ** *p* < 0.01; *** *p* < 0.001; ns = not significant). Luciferase values were corrected for protein concentration of the sample and the corrected value for the empty vector was arbitrarily set as 100%.

The LTag of polyomaviruses works as an activator and repressor of the early and late viral promoter, depending on the concentration. At low concentrations, LTag will activate the early promoter and thereby stimulate its own expression. At the same time, it will repress the late promoter. At higher concentrations, LTag represses the early promoter and activates the late promoter (reviewed in [39]). Therefore, we wanted to perform dose-response studies with different concentrations of LTag (respectively MKL2) expression plasmids. HaCaT cells were co-transfected with the luciferase reporter plasmid with the mutE or mutL promoter, and a possible dose-dependent effect of LTag was investigated, by co-transfecting with either 100 ng or 400 ng expression plasmid, for LTag. Higher concentrations were not examined because our previous studies had shown promoter interference between the stronger CMV (cytomegalovirus) promoter which drives LTag expression and the MCPyV of early and late promoters [40]. Both the early and late promoter activity decreased in the presence of full-length LTag. Single mutants replacing serine by non-phosphorylable alanine (A) or phosphomimicking aspartic acid (D) also significantly inhibited early and late promoter activity. No significant differences were found between wild-type full-length LTag and the mutants, and between the mutants, except for LT-S203D, which was a stronger inhibitor than LT-S203A on the early promoter (Figure 4). Western blot results showed that the non-mutated and single mutants of LTag were expressed at comparable levels (Supplementary Figure S3A).

**Figure 5:**
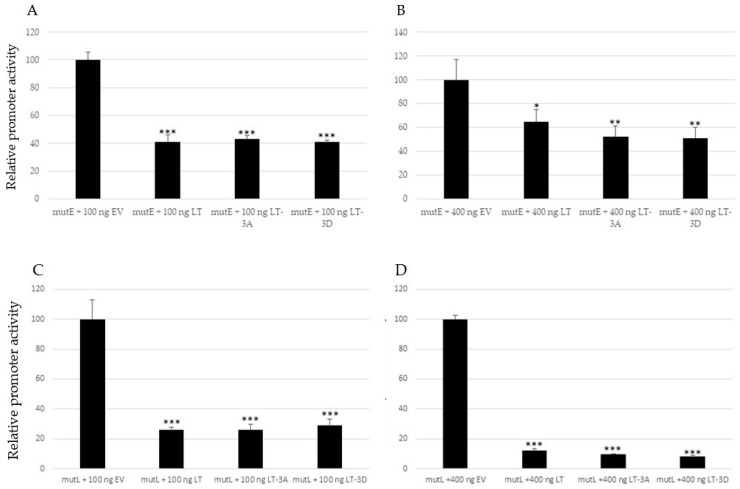
Full-length LTag and triple mutants inhibit MCPyV early and late promoter activity. HaCaT cells were co-transfected with 1 μg luciferase reporter plasmid containing the replication deficient early (**A**,**B**), or late promoter (**C**,**D**) and 100 ng (**A**,**C**), or 400 ng (**B**,**D**) empty vector (EV), or expression plasmid for full-length LTag, the tripe mutants 3A (LT-3A) or 3D (LT-3D). Each bar represents the average of three independent parallels with standard deviation. Statistically significant differences with empty expression vector are indicated (* *p* < 0.05; ** *p* < 0.01; and *** *p* < 0.001). Luciferase values were corrected for protein concentration of the sample and the corrected value for the empty vector was arbitrarily set as 100%.

Similarly, LTag with triple non-phosphorylable mutations (LT-3A) or phoshphomimicking substitutions (LT-3D) inhibited MCPyV early and late promoter activity, and no significant difference between wild-type LTag and the triple mutants and between 3LT-A and LT-3D, respectively, was measured (Figure 5). Western blot analysis demonstrated that non-mutated and triple mutated LTag are expressed at similar levels (Supplementary Figure S4).

### 2.4. Non-Phosphorylable and Phosphomimicking Substitutions of the PKA Phosphoacceptor Sites Have Opposite Effect on MKL2 LTag Induced Activation of the MCPyV Promoters

**Figure 6:**
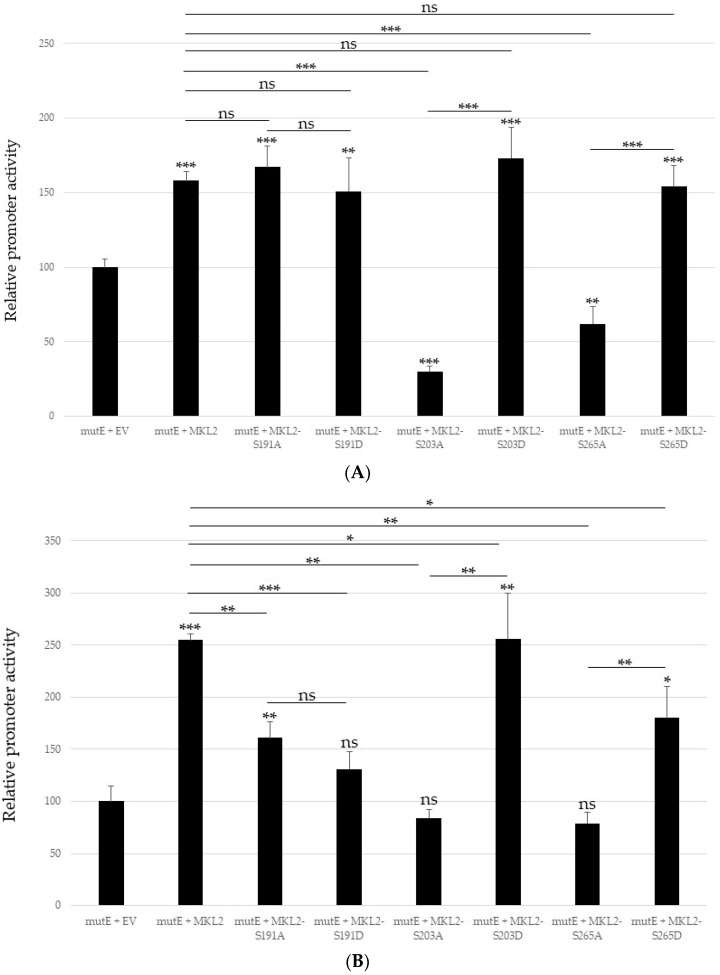

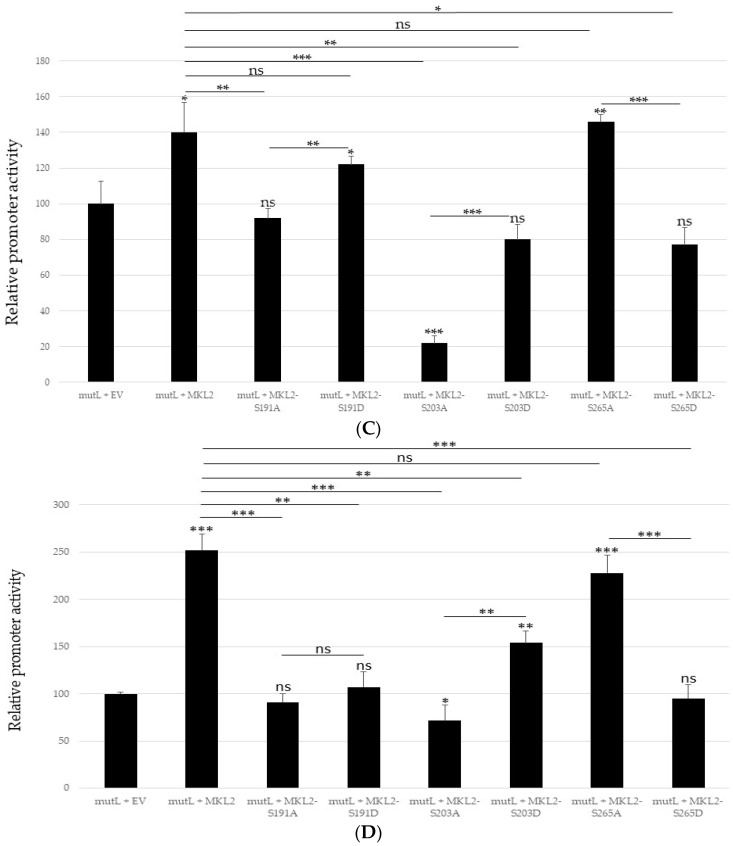
Single substitutions in PKA phosphoacceptor residues alter MKL2 LTag’s effect on MCPyV early and late promoter. Cells were co-transfected with luciferase reporter plasmid containing the replication deficient early (**A**,**B**) or late promoter (**C**,**D**), and 100 ng (**A**,**C**) or 400 ng (**B**,**D**) empty vector (EV), or expression plasmid for MKL2 LTag or single mutants in the residues S191, S203, and S265, respectively. Statistically significant differences with empty expression vector are indicated on top of the bar, whereas differences between MKL2 LTag and the mutants and between the A and D mutants are indicated on top of the horizontal lines (* *p* < 0.05; ** *p* < 0.01; and *** *p* < 0.001; ns = not significant). Luciferase values were corrected for protein concentration of the sample, and the corrected value for the empty vector was arbitrarily set as 100%.

Because MCPyV-positive MCCs express C-terminal truncated LTag, we examined the role of the putative PKA sites in the truncated LTag variant expressed in the virus-positive MCC cell line MKL2. Single mutations with the replacement S203A and S265A reduced transactivation of the MCPyV early promoter by MKLK2LTag, whereas substitutions S203D and S265D restored the induction of the early promoter by MKL2 LTag. Mutations of S191 did not affect stimulation of the early promoter by MKL2 LTag (Figure 6A). However, at higher concentrations of transfected plasmid (400 ng versus 100 ng), both the S191A and S191D partially reduced transactivation of the early promoter, compared to non-mutated MKL2 LTag (Figure 6B). MKL2 LTag stimulated MCPyV late promoter activity, whereas the MKL2-S191A and the MKL2-S203A mutants exerted an inhibitory effect on this promoter (Figure 6C,D). The S265A MKL2 LTag mutant retained the ability to stimulate the late promoter. S191D and S265D MKL2 LTag mutants reduced late promoter activity compared to MKL2 LTag, whereas the phosphomimicking S203D mutants behaved as non-mutated MKL2 LTag, and restored the inhibitory effect observed for MKL2-S203A (Figure 6C,D).

**Figure 7:**
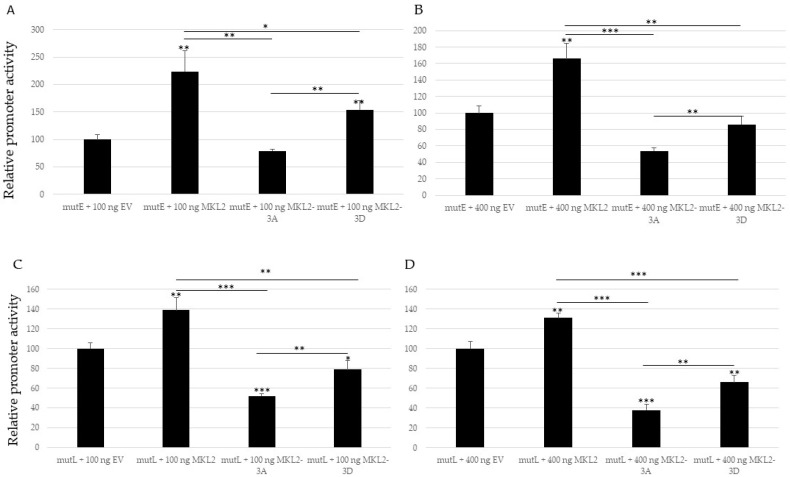
Alanine substitutions abrogate whereas aspartic acid replacements of the PKA phosphoacceptor residues restore MKL2 LTag’s ability to induce the MCPyV early and late promoter. HaCaT cells were co-transfected with a luciferase plasmid containing the non-replicative early promoter (mutE) and 100 ng (**A**) or 400 ng (**B**) expression plasmids. (**C**) and (**D**) as (**A**) and (**B**), respectively, but cells were transfected with a luciferase plasmid containing the MCPyV late promoter (mutL). Each bar represents the average of three independent parallels with standard deviation. Statistically significant differences with empty expression vector are indicated on top of the bar, whereas differences between MKL2 LTag and the mutants and between the 3A and 3D mutant are indicated on top of the horizontal lines (* *p* < 0.05; ** *p* < 0.01; and *** *p* < 0.001. Luciferase values were corrected for the protein concentration of the sample, and the corrected value for the empty vector was arbitrarily set as 100%.

Next, we tested the effect of MKL2 LTag with triple mutations. Simultaneous substitution of S191, S203, and S265 into Ala (MKL2-3A) abrogated transactivation of the early and late promoter, whereas triple Asp substitutions (MKL2-3D) partially restored MKL2 LTag’s ability to stimulate the MCPyV early and late promoter (Figure 7).

### 2.5. Mutations in PKA Phosphoacceptor Sites of MKL2 LTag Affect Transactivation of the Cellular Promoters CCL17 and IL33

**Figure 8:**
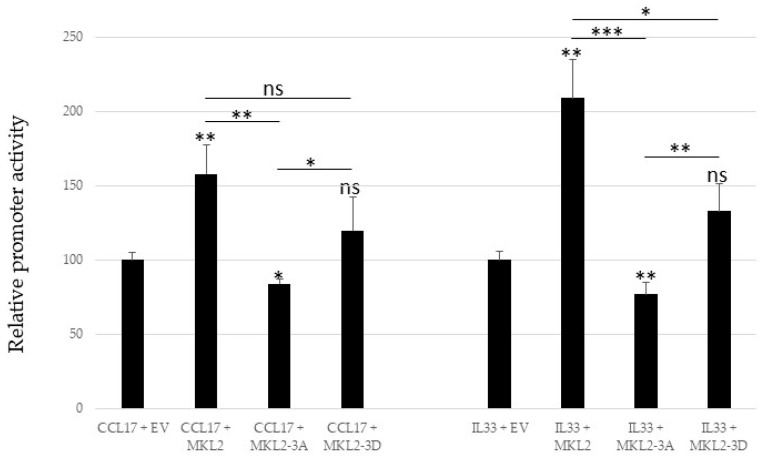
Mutations in the PKA phosphoaccpetor sites of MKL2 LTag affect transactivation of the CCL17 and IL33 promoters. Cells were co-transfected with a luciferase reporter plasmid containing the sequences −2535/+40 of the CCL17 promoter or −1050/+45 of the IL33 promoter, and empty expression vector (EV) or expression vector for MKL2, MKL2-3A, or MKL2-3D LTag. The bars represent the average of three independent parallels ± SD. The statistically significant difference between promoter activity in the presence of EV and MKL2 (respectively MKL2-3A and MKL2-3D) is shown on top of the bars, whereas differences between MKL2 and the mutants and between MKL2-3A and MKL2-3D are given on top of the lines. * *p* < 0.05, ** *p* < 0.01, and *** *p* < 0.001, ns = not significant.

We had previously shown that the expression of CCL17 and IL33 is enhanced in MCPyV-positive MCC tumors and MCC cell lines (including MKL2 MCC cells), compared to virus-negative tumors and cell-lines, and that the CCL17 and IL33 promoters are activated by MCPyV MKL2 [41,42]. This prompted us to examine whether mutations in the PKA sites influence MKL2 LTag to transactivate cellular promoters. Expression of MKL2 LTag transactivated the CCL17 and IL33 promoters in HaCaT cells (Figure 8). The triple MKL2-3A mutation abrogated transactivation, whereas the phosphomimicking MKL2-3D mutant partially restored transactivation of both promoters. These results indicate that PKA-mediated phosphorylation regulates the transcriptional activity of MKL2 LTag.

### 2.6. Activation of the cAMP-Dependent Protein Kinase Pathway Does Not Affect MCPyV Promoter Activity

**Figure 9:**
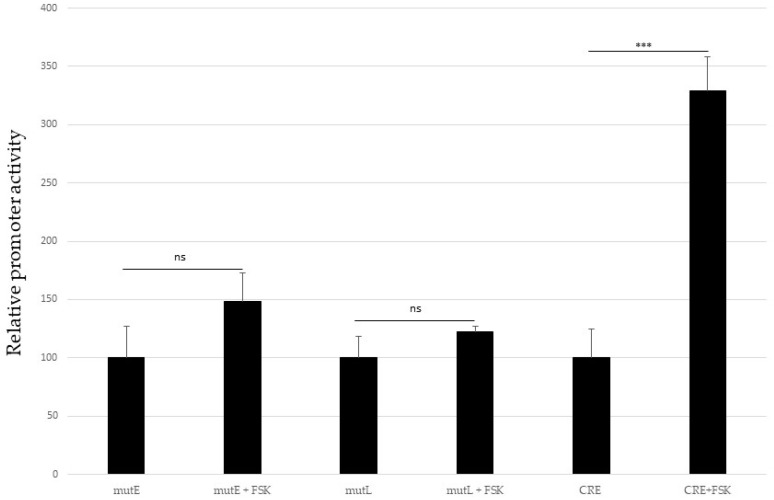
Activation of the PKA pathway does not induce the MCPyV early and late promoter. HaCaT cells were transfected with a luciferase reporter plasmid containing either the MCPyV early or late promoter, or a minimal promoter with four copies of the cAMP response element consensus sequence (CRE). Cells were serum-starved for 24 h and then treated with 10 μM forskolin (FSK) for 1 h. Luciferase activity was corrected for protein concentration in each sample, and promoter activity in the absence of forskloin was set as 100%. Each bar is the average of three independent parallels ± SD. The statistical significance between non-treated and forskolin treated cells is shown with ns = not significant and *** *p* < 0.001. The results of a representative experiment are shown.

The observation that putative PKA phosphorylation sites in MKL2 LTag have an impact on its transcriptional activity urged us to test whether expression of LTag can be regulated by PKA. Therefore, we examined whether the MCPyV promoter is cAMP-responsive. Transfected HaCaT cells were serum-starved for 24 h and then exposed to 10 μM forskolin, an activator of adenylyl cyclase [43]. One hour later, cells were harvested, and luciferase activity was measured. Neither the early nor the late promoter were induced by forskolin, whereas a minimal promoter with cAMP response elements (CRE) was stimulated approximately 3-fold (Figure 9).

### 2.7. Activation of the PKA Pathway Does Not Affect LTag Expression Levels

**Figure 10:**
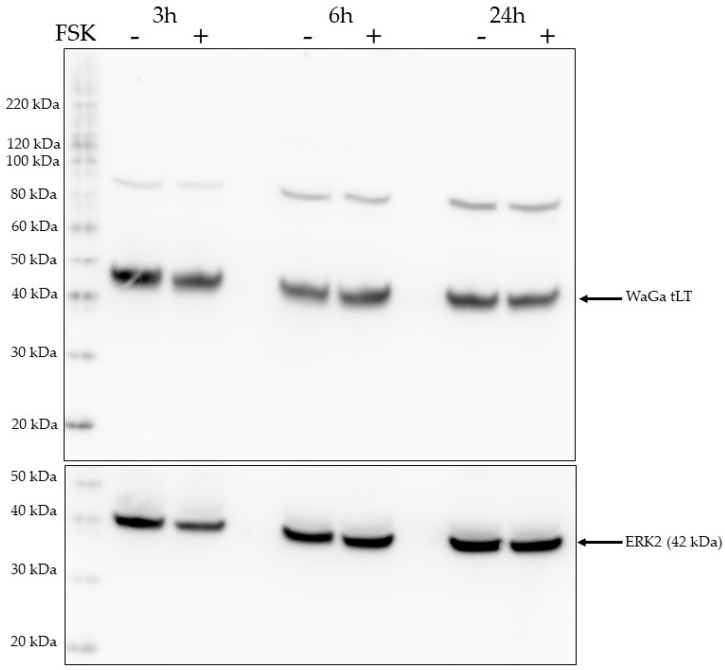
Activation of the PKA pathway does not affect LTag expression levels in the MCC cell line WaGa. Cells were serum-starved for 18 h and then exposed to 10 mM forskolin for the time points shown. WaGa cells express a truncated LTag of approximately 45 kDa. ERK2 was used as a loading control. The molecular marker is shown in the lane on the left.

The MCPyV-positive MCC cell line WaGa was treated with forskolin. Cells were serum-starved for 18 h and then exposed to 10 μM forskolin. MCPyV LTag expression levels were analyzed 30 min, 60 min, 180 min, 360 min, and 24 h after forskolin treatment. No increase in LTag levels was observed compared to mock treated cells for any of the time points (Figure 10 and results not shown). ERK2 was used as a loading control because it has been shown to be a reliable control protein for equal protein loading, and ERK2 expression levels are not affected by forskolin [44,45,46]. Similar results were obtained in MS1 cells, another virus positive MCC cell line. No change in MS1 LTag levels were observed in these cells treated for 30 min, 60 min, 180 min, 360 min, and 24 h (Supplementary Figure S5).

## 3. Discussion

LTag of polyomaviruses is indispensable for genome replication but is also necessary for expression of their genes [3]. Phosphorylation has been shown to affect these activities for the LTag of the polyomaviruses SV40, JC, and murine polyomavirus [47,48,49,50]. Several phosphorylation sites have been identified in MCPyV LTag (Supplementary Figure S1), but the protein kinases that mediate these phosphorylations remain largely unidentified, nor has the effect on LTag’s transcriptional activity been examined. Using the NetPhos 3.1 program to predict PKA phosphorylation site, we identified three putative PKA phosphorylation sites in MCPyV LTag (S191, S203, and S265), and confirmed in vitro phosphorylation of S203 and S265 by mass spectrometry. Studies with mutants in these sites altered LTag’s effect on the early and late MCPyV promoter. As previously shown in HEK293 cells, human dermal fibroblasts, and the MCPyV-negative MCC line MCC13 cells, full-length LTag inhibited both the MCPyV early and late promoter [10,35]. The truncated LTag variant MKL2, however, modestly stimulated the MCPyV promoters. The conversion of full-length LTag, as a repressor, into MKL2, as a transcriptional activator, results from the deletion of the C-terminal region, which contains the DNA binding domain. The mechanism by which MKL2 activates the MCPyV promoters is not known, but may involve binding of cellular transcriptional activating proteins, that are not recruited by full-length LTag. The difference in transcriptional activity of LTag and MKL2 will translate in different concentrations of these proteins. LTag autorepresses its own expression, whereas MKL2 will enhance its own expression. The higher expression levels of MKL2 may therefore be high enough to inhibit retinoblastoma proteins and interfere with other cellular processes required for transformation. Moreover, increased transcriptional activity of MKL2 LTag stimulated the activity of the CCL17 and IL-33 promoters (1.5 and 2.0-fold, respectively). Our previous studies have shown that these cytokines are highly expressed in MCPyV-positive MCC, but their role in MCC remains unsolved [41,42]. However, CCL17 and IL-33 are involved in different cancers [51,52,53].

Mutations that prevent phosphorylation (S into A) or that mimic phosphorylation (S into D) of the residues 191, 203, and 265 did not alter the transcription repression of both promoters. Kwun and colleges observed similar results for S147A, S220A, and S293 mutants. Mutants in which these residues were replaced by D were not examined [10]. We also examined the transcription activity of a truncated LTag which is expressed in virus-positive MCC tumors. The truncated MKL2 Ltag variant stimulated MCPyV early and late promoter activity in HaCaT cells. This is in accordance with our previous results which showed that truncated Ltag MKL1 and MS1 increased the activity of the MCPyV promoters in human dermal fibroblast and MCC13 cells [35]. It is not known why full-length Ltag hampers, whereas truncated increases MCPyV promoter activity. All sequenced truncated Ltag isolated from MCC tissue or cells lack their DNA binding domain, preventing them from binding viral DNA. The viral DNA:LTag interaction may negatively interfere with MCPyV promoter activity. Another possibility is that truncated and full-length LTag bind with different cellular proteins. These interactions may turn full-length LT into a repressor complex, whereas truncated LTag may form a transcriptional activation complex. A different conformation than full-length LTag and phosphorylation of truncated LTag may also influence the interaction with other proteins. A biological consequence of invigorating early promoter activity by truncated LTag is the increase of its own expression levels, as well as that of the other oncoprotein sTag, which may contribute to the development of MCC.

Although proteomic analysis failed to detect phosphorylation of S191 (this study and [12]), substitution of this residue into Ala or Asp altered the transcriptional activity of MKL2 LTag. It is possible that this residue is not a bona fide PKA phosphoacceptor site, but that the mutation induces a conformational change or affects binding of its interaction partner, resulting in altered activity of the protein. The lack of detection of S191 phosphorylation could also be due to other reasons such as the sensitivity of the method, the inability of PKA to bind (we used an oligopeptide with N-terminal location of S191), or inaccessibility of S191 because of the conformation of the truncated LTag studied by Schrama and his colleges [12]. Finally, phosphorylation of S265 may be required before S191 becomes phosphorylated, which could not be achieved in our study because peptide 1 contained S191 and S203, but lacked S265. Sequential phosphorylation whereby one phosphorylation primes the phosphorylation of another site has been reported for other proteins [54,55,56,57].

The effect of mutations in the PKA phosphoacceptor sites of MCPyV LTag on the interaction cellular proteins, including retinoblastoma protein, hVam6p, Skp2, and on other cellular processes such as proliferation, apoptosis, cell mobility, and invasion remains to be examined. Xiao et al. showed that introducing a PKA site in SV40 LTag by site directed mutagenesis stimulated the rate of nuclear import of LTag [58]. Another study using a MCPyV truncated LTag with the mutations S246A/S247A/S254A/T257A/S265A/T271A did not alter nuclear import of the protein [12]. This suggests that S265 is not involved in subcellular localization of truncated LTag. We have not examined whether mutations in the PKA sites influenced subcellular localization of full-length or truncated LTag. MKL2 LTag has a molecular mass < 60 kDa, which would allow passive diffusion into the nucleus without the requirement of a nuclear localization signal and the importin system [59]. Therefore, PKA may not regulate their nuclear entrance. In vivo phosphorylation of these sites has not been confirmed, but development of phosphospecific antibodies may be a technical challenge due to the amino acid sequence of LTag with overrepresentation and consecutive occurrence of serine residues.

## 4. Materials and Methods

### 4.1. Cells

The human keratinocyte cell line HaCaT was purchased from Cell Lines Services (Eppelheim, Germany, cat. No. 300493) and maintained in Dulbecco’s modified Eagle’s medium (Sigma-Aldrich, Saint-Louis, MO, USA, cat. No. D5796), with 10% fetal bovine serum (FBS; Life Technologies Limited, Pailey, UK), in the presence of 100 μg/mL streptomycin and 100 units/mL penicillin. Immortalized human dermal fibroblasts fHDF/TERT166 (Evercyte, Vienna, Austria, cat. no. CHT-031-0166) were kept in DMEM/Ham’s F12 (1:1) (Biochrom, Berlin, Germany; cat. no. F4815), 10% FBS, 2mM GlutaMaxTM-I (Gibco; cat. no. 35050-038) and 100 μg/mL G418 (Santa Cruz Biotechnology, Dallas, TX, USA; cat. no. sc-29065). The MCPyV-positive cell lines MS1 and WaGa were grown in RPMI medium (Sigma-Aldrich), with 10% FBS and 100 μg/mL streptomycin and 100 units/mL penicillin [60,61]. The WaGa cell line was a kind gift from Dr. JC Becker (University Duisburg-Essen, Germany). Cells were kept in a humidified CO_2_ incubator at 37 °C. Opti-Mem^TM^ was obtained from ThermoFisher Scientific (Waltham, MA, USA, cat. no. 31985070).

### 4.2. Plasmids

The luciferase reporter plasmid with the consensus NCCR of MCPyV in early (pGL3-cons-E) and late (pGL3-cons-L) orientation have been previously described [62]. These plasmids were used to make non-replicating competent plasmids by a double point mutation in a LTag binding motif in the NCCR, which has been demonstrated to prevent LTag-mediated replication [10]. In this work, these plasmids are referred to as pmutE-LUC and pmutL-LUC, respectively. The empty expression vector pcDNA3.1(+) was purchased from Invitrogen (ThermoFisher Scientific) and the pCRE-LUC vector was from Stratagene (San Diego, CA, USA). The MCPyV expression vectors for full-length LTag and truncated MKL2 and MS1 LTag, and the luciferase reporter plasmid containing the CCL17 promoter fragment, spanning nucleotides −2535/+40 or the IL33 −1050/+50 promoter sequence, have been previously described [39,40]. Site-directed mutagenesis was used to generate plasmids expressing mutants of LTag and MKL2. For the triple MKL2 LTag mutants, single mutants were made, subsequently double mutants, and finally triple mutants. Site-directed mutagenesis was used to introduce a stop codon in the expression plasmids for full-length LTag and its single mutants S191A, S191D, S203A, and S203D, thereby creating expression vectors for MCC350 LT and its single mutants. All primers used in site-directed mutagenesis were obtained from Sigma-Aldrich and are given in Supplementary Table S3. All mutations were verified by sequencing.

### 4.3. Forskolin Stimulation

Cells were serum starved for 18 h by keeping them in Opti-MeM^TM^ medium with 0.5% human albumin (Albunorm Octapharma, Vienna, Austria). Forskolin (Sigma-Aldrich; cat. no. F6886) was added to a final concentration of 10 μM, and cells were harvested at different time points, as mentioned in the result section.

### 4.4. Transfection and Luciferase Assay

HaCaT cells were seeded out in 12-well culture plates. At the time of transfection, the cells were approximately 70% confluent. A total of 1 μg luciferase reporter plasmid DNA was used per well and polyethylenimine (PEI linear MW25,000, transfection grade, cat. no. 23966-1, Polysciences, Warrington, PA, USA). DNA was mixed with 150 mM NaCl and the mixture of DNA:150 mM NaCl was then added to PEI:150 mM NaCl. The ratio DNA:PEI used was 1:2. This mixture was left for 15 min at room temperature and then carefully added to the cells. The medium containing the transfection mixture was replaced 4 h later. Cells were harvested 24 h after transfection in 100 μL Tropix lysis buffer per well, with 0.5 mM DTT freshly added. Cells were centrifuged for 3 min at 12,000 g and the supernatant was transferred to a fresh tube. Twenty μL of the supernatant were added to a 96-well microtiter plate containing 50 μL luciferase buffer (Promega, Madison, WI, USA). The CLARIOstar Plus Microplate reader (BMG Labtech, Ortenberg, Germany) was used to measure relative luciferase units (RLU). Each experiment was repeated 2–4 times with three independent parallel samples for each experiment. Luciferase values were corrected for the total protein concentration, which was measured using the MN protein quantification assay (Macherey-Nagel GmbH, Düren, Germany). OD570 was measured using the CLARIONstar Plus Microplate reader. Human dermal fibroblasts were transfecting with JetPrime (Polyplus, Illkirch, France) according to the manufacturer’s instructions, and similar results were obtained.

### 4.5. Western Blot

Western blot was performed by separating protein samples on 4–12% NuPAGE Bis-Tris Mini Gels (Invitrogen Life Technologies, Carlsbad, CA, USA), according to the manufacturer’s protocol. Proteins were blotted onto a 0.45 μm PVDF membrane (Millipore, Billerica, MA, USA), and blocking was performed using TBST (TBS with 0.1% Tween-20; Sigma-Aldrich) containing 5% (*w*/*v*) dried skimmed milk for 1 h. The protein was probed by incubating the membrane with the primary antibody overnight at 4 °C. After washing the membrane 3 times with TBST, an appropriate secondary antibody was added for 1 h at room temperature. After 2 washes with TBST and 2 washes with washing buffer, antigen-antibody complex was visualized using SuperSignal™ West Pico Chemiluminescent Substrate (cat.no. 34080 Thermo Fisher Scientific, Rockford, IL, USA). The Magic-Mark™ Western standard from Invitrogen Life Technologies was used to estimate the molecular mass of the detected proteins. For the detection of MCPyV LTag, CM2B4 (Santa Cruz Biotechnology, Dallas, TX, USA; cat. no. sc-136172). ERK2 and GADPH antibodies were from Santa Cruz (cat. no. sc-154 and sc-47724, respectively).

### 4.6. In Vitro Kinase Assay

The peptides GRESSTPNGTSVPRNSSRTDGTWEDLFCDE (residues 187–216 in MKL2 LTag) and QFTDEEYRFSSFTTPKTPPAF (residues 255–275 in MKL2 LTag) were purchased from GenScript Biotech (Leiden, The Netherlands). The catalytic subunit of PKA was obtained from Promega (Madison, WI, USA, cat. no. V516A). In vitro kinase assay was performed as previously outlined, except that no radioactive [γ-^32^P]-ATP was used [63].

### 4.7. Mass Spectrometry

Peptides containing 0.1% formic acid were loaded onto a Thermo Fisher Scientific EASY-nLC1200 system. Peptides were separated using a C18 nano column (EASY-Spray column, C18, 2 μm, 100 Å, 50 μm, 50 cm; ThermoFisher). Peptides were fractionated using a 4–40% gradient of increasing amounts of 80% Acetonitrile in water for over 30 min at a flow rate of 300 nl/min. The mobile phases contained 0.1% formic acid. Peptides were analyzed using an Orbitrap Exploris 480 mass spectrometer. MS1 resolution was set to 45,000 while MS2 resolution was 60,000. HCD collision energy of 34 and 40% was used. Protein identification and PTM mapping was done using the Proteome Discoverer 2.5 software (ThermoFisher) using the ptmRS module (>75%).

### 4.8. Statistics

Luciferase values were corrected for protein concentration in the sample and the average of the three independent parallels ±SD was calculated. An unpaired *t*-test from GraphPad (San Diego, CA, USA) was used to determine whether differences were significant (*p* < 0.05).

## 5. Conclusions

Substitution of serine residues 191, 203 and 265 into non-phosphorylable Ala or phosphomimicking Asp did not alter the transcriptional repression activity of MCPyV LTag on the viral promoters. Truncated MKL2 LTag activated the viral promoters and the promoters of the cytokines CCL17 and IL-33, in a PKA-phophorylation dependent manner. PKA-dependent regulation of the transcriptional activity of truncated LTag, which is expressed in MCC, may therefore contribute to tumorigenesis. CISPR/Cas9 technology can be used to introduce the S203A mutation in the PKA site of truncated LTag, expressed in virus positive MCC tumors, to repress the activation of the MCPyV early promoter, therefore abrogating expression of the oncoproteins LTag and sTag. Alternatively, overexpression of truncated Ltag S203A mutant may inhibit early promoter activity and hence hamper expression of endogenous Ltag and sTag.

## Data Availability

The data presented in this study are available upon request from the corresponding author.

## Supplementary Materials

The following supporting information can be downloaded at: https://www.mdpi.com/article/10.3390/ijms24010895/s1, ref. [64] in Supplementary Materials.

## References

[1] Feng H., Shuda M., Chang Y., Moore P.S. (2008). Clonal integration of a polyomavirus in human Merkel cell carcinoma. Science.

[2] Becker J.C., Stang A., DeCaprio J.A., Cerroni L., Lebbé C., Veness M., Nghiem P. (2017). Merkel cell carcinoma. Nat. Rev. Dis. Prim..

[3] Moens U., Krumbholz A., Ehlers B., Zell R., Johne R., Calvignac-Spencer S., Lauber C. (2017). Biology, evolution, and medical importance of polyomaviruses: An update. Infect. Genet. Evol..

[4] Schowalter R.M., Buck C.B. (2013). The Merkel cell polyomavirus minor capsid protein. PLoS Pathog..

[5] DeCaprio J.A., Garcea R.L. (2013). A cornucopia of human polyomaviruses. Nat. Rev. Microbiol..

[6] Chang Y., Moore P.S. (2012). Merkel cell carcinoma: A virus-induced human cancer. Annu. Rev. Pathol..

[7] Pietropaolo V., Prezioso C., Moens U. (2020). Merkel Cell Polyomavirus and Merkel Cell Carcinoma. Cancers.

[8] De Caprio J.A. (2021). Molecular Pathogenesis of Merkel Cell Carcinoma. Annu. Rev. Pathol..

[9] Schlemeyer T., Ohnezeit D., Virdi S., Körner C., Weißelberg S., Starzonek S., Schumacher U., Grundhoff A., Indenbirken D., Albertini S. (2022). Merkel cell carcinoma and immune evasion: Merkel cell polyomavirus small T-antigen induced surface changes can be reverted by therapeutic intervention. J. Investig. Dermatol..

[10] Kwun H.J., Chang Y., Moore P.S. (2017). Protein-mediated viral latency is a novel mechanism for Merkel cell polyomavirus persistence. Proc. Natl. Acad. Sci. USA.

[11] Nwogu N., Ortiz L.E., Kwun H.J. (2020). Merkel Cell Polyomavirus Large T Antigen Unique Domain Regulates Its Own Protein Stability and Cell Growth. Viruses.

[12] Schrama D., Hesbacher S., Angermeyer S., Schlosser A., Haferkamp S., Aue A., Adam C., Weber A., Schmidt M., Houben R. (2016). Serine 220 phosphorylation of the Merkel cell polyomavirus large T antigen crucially supports growth of Merkel cell carcinoma cells. Int. J. Cancer.

[13] Diaz J., Wang X., Tsang S.H., Jiao J., You J. (2014). Phosphorylation of large T antigen regulates merkel cell polyomavirus replication. Cancers.

[14] Li J., Diaz J., Wang X., Tsang S.H., You J. (2015). Phosphorylation of Merkel cell polyomavirus large tumor antigen at serine 816 by ATM kinase induces apoptosis in host cells. J. Biol. Chem..

[15] Alvarez Orellana J., Kwun H.J., Artusi S., Chang Y., Moore P.S. (2021). Sirolimus and Other Mechanistic Target of Rapamycin Inhibitors Directly Activate Latent Pathogenic Human Polyomavirus Replication. J. Infect. Dis..

[16] Cohen P. (2000). The regulation of protein function by multisite phosphorylation—A 25 year update. Trends Biochem. Sci..

[17] Pawson T., Scott J.D. (2005). Protein phosphorylation in signaling—50 years and counting. Trends Biochem. Sci..

[18] Ardito F., Giuliani M., Perrone D., Troiano G., Lo Muzio L. (2017). The crucial role of protein phosphorylation in cell signaling and its use as targeted therapy (Review). Int. J. Mol. Med..

[19] Cargnello M., Roux P.P. (2011). Activation and function of the MAPKs and their substrates, the MAPK-activated protein kinases [published correction appears in *Microbiol. Mol. Biol. Rev.* **2012**, *76*, 496]. Microbiol. Mol. Biol. Rev..

[20] Trenker R., Jura N. (2020). Receptor tyrosine kinase activation: From the ligand perspective. Curr. Opin. Cell Biol..

[21] Pinna L.A., Ruzzene M. (1996). How do protein kinases recognize their substrates?. Biochim. Biophys. Acta.

[22] Mayr B., Montminy M. (2001). Transcriptional regulation by the phosphorylation-dependent factor CREB. Nat. Rev. Mol. Cell Biol..

[23] Johannessen M., Delghandi M.P., Moens U. (2004). What turns CREB on?. Cell Signal.

[24] Moens U., Sundsfjord A., Flaegstad T., Traavik T. (1990). BK virus early RNA transcripts in stably transformed cells: Enhanced levels induced by dibutyryl cyclic AMP, forskolin and 12-O-tetradecanoylphorbol-13-acetate treatment. J. Gen. Virol..

[25] Love T.M., de Jesus R., Kean J.A., Sheng Q., Leger A., Schaffhausen B. (2005). Activation of CREB/ATF sites by polyomavirus large T antigen. J. Virol..

[26] Olsen J.V., Blagoev B., Gnad F., Macek B., Kumar C., Mortensen P., Mann M. (2006). Global, in vivo, and site-specific phosphorylation dynamics in signaling networks. Cell.

[27] Smith F.D., Samelson B.K., Scott J.D. (2011). Discovery of cellular substrates for protein kinase A using a peptide array screening protocol. Biochem. J..

[28] Imamura H., Wagih O., Niinae T., Sugiyama N., Beltrao P., Ishihama Y. (2017). Identifications of Putative PKA Substrates with Quantitative Phosphoproteomics and Primary-Sequence-Based Scoring. J. Proteome Res..

[29] Isobe K., Jung H.J., Yang C.R., Claxton J., Sandoval P., Burg M.B., Raghuram V., Knepper M.A. (2017). Systems-level identification of PKA-dependent signaling in epithelial cells. Proc. Natl. Acad. Sci. USA.

[30] Karamafrooz A., Brennan J., Thomas D.D., Parker L.L. (2021). Integrated Phosphoproteomics for Identifying Substrates of Human Protein Kinase A (PRKACA) and Its Oncogenic Mutant DNAJB1-PRKACA. J. Proteome Res..

[31] Blom N., Gammeltoft S., Brunak S. (1999). Sequence and structure-based prediction of eukaryotic protein phosphorylation sites. J. Mol. Biol..

[32] Hashida Y., Imajoh M., Kamioka M., Taniguchi A., Kuroda N., Hayashi K., Nakajima H., Sano S., Daibata M. (2014). Phylogenetic analysis of Merkel cell polyomavirus based on full-length LT and VP1 gene sequences derived from neoplastic tumours in Japanese patients. J. Gen. Virol..

[33] Matsushita M., Iwasaki T., Kuwamoto S., Kato M., Nagata K., Murakami I., Kitamura Y., Hayashi K. (2014). Merkel cell polyomavirus (MCPyV) strains in Japanese merkel cell carcinomas (MCC) are distinct from Caucasian type MCPyVs: Genetic variability and phylogeny of MCPyV genomes obtained from Japanese MCPyV-infected MCCs. Virus Genes.

[34] Wu Z., Jin Y., Chen B., Gugger M.K., Wilkinson-Johnson C.L., Tiambeng T.N., Jin S., Ge Y. (2019). Comprehensive Characterization of the Recombinant Catalytic Subunit of cAMP-Dependent Protein Kinase by Top-Down Mass Spectrometry. J. Am. Soc. Mass Spectrom..

[35] Abdulsalam I., Rasheed K., Sveinbjørnsson B., Ehlers B., Moens U. (2020). Promoter activity of Merkel cell Polyomavirus variants in human dermal fibroblasts and a Merkel cell carcinoma cell line. Virol. J..

[36] Boukamp P., Petrussevska R.T., Breitkreutz D., Hornung J., Markham A., Fusenig N.E. (1988). Normal keratinization in a spontaneously immortalized aneuploid human keratinocyte cell line. J. Cell Biol..

[37] Sunshine J.C., Jahchan N.S., Sage J., Choi J. (2018). Are there multiple cells of origin of Merkel cell carcinoma?. Oncogene.

[38] Kervarrec T., Samimi M., Guyétant S., Sarma B., Chéret J., Blanchard E., Berthon P., Schrama D., Houben R., Touzé A. (2019). Histogenesis of Merkel Cell Carcinoma: A Comprehensive Review. Front. Oncol..

[39] Farmerie W.G., Folk W.R. (1984). Regulation of polyomavirus transcription by large tumor antigen. Proc. Natl. Acad. Sci. USA.

[40] Rasheed K., Sveinbjørnsson B., Moens U. (2021). Reciprocal transactivation of Merkel cell polyomavirus and high-risk human papillomavirus promoter activities and increased expression of their oncoproteins. Virol. J..

[41] Rasheed K., Moens U., Policastro B., Johnsen J.I., Koljonen V., Sihto H., Lui W.O., Sveinbjørnsson B. (2022). The Merkel Cell Polyomavirus T-Antigens and IL-33/ST2-IL1RAcP Axis: Possible Role in Merkel Cell Carcinoma. Int. J. Mol. Sci..

[42] Rasheed K., Abdulsalam I., Fismen S., Grimstad Ø., Sveinbjørnsson B., Moens U. (2018). CCL17/TARC and CCR4 expression in Merkel cell carcinoma. Oncotarget.

[43] Seamon K.B., Padgett W., Daly J.W. (1981). Forskolin: Unique diterpene activator of adenylate cyclase in membranes and in intact cells. Proc. Natl. Acad. Sci. USA.

[44] Zanassi P., Paolillo M., Feliciello A., Avvedimento E.V., Gallo V., Schinelli S. (2001). cAMP-dependent protein kinase induces cAMP-response element-binding protein phosphorylation via an intracellular calcium release/ERK-dependent pathway in striatal neurons. J. Biol. Chem..

[45] Dittmer A., Dittmer J. (2006). Beta-actin is not a reliable loading control in Western blot analysis. Electrophoresis.

[46] Vuchak L.A., Tsygankova O.M., Prendergast G.V., Meinkoth J.L. (2009). Protein kinase A and B-Raf mediate extracellular signal-regulated kinase activation by thyrotropin. Mol. Pharmacol..

[47] Mohr I.J., Stillman B., Gluzman Y. (1987). Regulation of SV40 DNA replication by phosphorylation of T antigen. EMBO J..

[48] Bockus B.J., Schaffhausen B. (1987). Phosphorylation of polyomavirus large T antigen: Effects of viral mutations and cell growth state. J. Virol..

[49] Swenson J.J., Frisque R.J. (1995). Biochemical characterization and localization of JC virus large T antigen phosphorylation domains. Virology.

[50] Swenson J.J., Trowbridge P.W., Frisque R.J. (1996). Replication activity of JC virus large T antigen phosphorylation and zinc finger domain mutants. J. Neurovirol..

[51] Larsen K.M., Minaya M.K., Vaish V., Peña M.M.O. (2018). The Role of IL-33/ST2 Pathway in Tumorigenesis. Int. J. Mol. Sci..

[52] Hong J., Kim S., Lin P.C. (2019). Interleukin-33 and ST2 Signaling in Tumor Microenvironment. J. Interferon Cytokine Res..

[53] Korbecki J., Kojder K., Simińska D., Bohatyrewicz R., Gutowska I., Chlubek D., Baranowska-Bosiacka I. (2020). CC Chemokines in a Tumor: A Review of Pro-Cancer and Anti-Cancer Properties of the Ligands of Receptors CCR1, CCR2, CCR3, and CCR4. Int. J. Mol. Sci..

[54] Schneider-Yin X., Gouya L., Dorsey M., Rüfenacht U., Deybach J.C., Ferreira G.C. (2000). Mutations in the iron-sulfur cluster ligands of the human ferrochelatase lead to erythropoietic protoporphyria. Blood.

[55] Tang Q.Q., Grønborg M., Huang H., Kim J.W., Otto T.C., Pandey A., Lane M.D. (2005). Sequential phosphorylation of CCAAT enhancer-binding protein beta by MAPK and glycogen synthase kinase 3beta is required for adipogenesis. Proc. Natl. Acad. Sci. USA.

[56] Huang G., Chen S., Li S., Cha J., Long C., Li L., He Q., Liu Y. (2007). Protein kinase A and casein kinases mediate sequential phosphorylation events in the circadian negative feedback loop. Genes Dev..

[57] Woo Y., Kim S.J., Suh B.K., Kwak Y., Jung H.J., Nhung T.T.M., Mun D.J., Hong J.H., Noh S.J., Kim S. (2019). Sequential phosphorylation of NDEL1 by the DYRK2-GSK3β complex is critical for neuronal morphogenesis. eLife.

[58] Xiao C.Y., Hübner S., Elliot R.M., Caon A., Jans D.A. (1996). A consensus cAMP-dependent protein kinase (PK-A) site in place of the CcN motif casein kinase II site simian virus 40 large T-antigen confers PK-A-mediated regulation of nuclear import. J. Biol. Chem..

[59] Timney B.L., Raveh B., Mironska R., Trivedi J.M., Kim S.J., Russel D., Wente S.R., Sali A., Rout M.P. (2016). Simple rules for passive diffusion through the nuclear pore complex. J. Cell Biol..

[60] Houben R., Shuda M., Weinkam R., Schrama D., Feng H., Chang Y., Moore P.S., Becker J.C. (2010). Merkel cell polyomavirus-infected Merkel cell carcinoma cells require expression of viral T antigens. J. Virol..

[61] Guastafierro A., Feng H., Thant M., Kirkwood J.M., Chang Y., Moore P.S., Shuda M. (2013). Characterization of an early passage Merkel cell polyomavirus-positive Merkel cell carcinoma cell line, MS-1, and its growth in NOD scid gamma mice. J. Virol. Methods.

[62] Moens U., Van Ghelue M., Ludvigsen M., Korup-Schulz S., Ehlers B. (2015). Early and late promoters of BK polyomavirus, Merkel cell polyomavirus, Trichodysplasia spinulosa-associated polyomavirus and human polyomavirus 12 are among the strongest of all known human polyomaviruses in 10 different cell lines. J. Gen. Virol..

[63] Van Lint J., Ni Y., Valius M., Merlevede W., Vandenheede J.R. (1998). Platelet-derived growth factor stimulates protein kinase D through the activation of phospholipase Cgamma and protein kinase C. J. Biol. Chem..

[64] Liu X., Hein J., Richardson S.C., Basse P.H., Toptan T., Moore P.S., Gjoerup O.V., Chang Y. (2011). Merkel cell polyomavirus large T antigen disrupts lysosome clustering by translocating human Vam6p from the cytoplasm to the nucleus. J. Biol. Chem..

