# Supplementary material for: Regulation of Transcriptional Activity of Merkel Cell Polyomavirus Large T-Antigen by PKA-Mediated Phosphorylation
Source: Int J Mol Sci. 2023 Jan 3;24(1):895. doi: 10.3390/ijms24010895 (PMC9820997; doi:10.3390/ijms24010895)
Supplement: Supplementary file 1 [file ijms-24-00895-s001.zip › ijms-2021411-supplementary.pdf]

International Journal of *Molecular Sciences*

Supplementary figures and tables

Regulation of Transcriptional Activity of Merkel Cell Polyomavirus Large T-Antigen by PKA-Mediated Phosphorylation

Mar Falquet [1], Carla Prezioso [2,3], Maria Ludvigsen [1], Jack-Ansgar Bruun [4], Sara Passerini [3], Baldur Sveinbjørnsson [1,5], Valeria Pietropaolo [3,*,†] and Ugo Moens [2,*,†]

1 Molecular Inflammation Research Group, Department of Medical Biology, University of Tromsø-The Arctic University of Norway, 9037 Tromsø, Norway
2 Microbiology of Chronic Neuro-Degenerative Pathologies, IRCSS San Raffaele, 00163 Rome, Italy
3 Department of Public Health and Infectious Diseases, Sapienza University of Rome, 00185 Rome, Italy
4 Department of Medical Biology, Proteomics Platform, University of Tromsø-The Arctic University of Noway, 9037 Tromsø, Norway
5 Childhood Cancer Research Unit, Department of Women's and Children's Health, Karolinska Institute, 17177 Stockholm, Sweden
* Correspondence: valeria.pietropaolo@uniroma1.it (V.P.); ugo.moens@uit.no (U.M.)
† These authors contributed equally to this work.

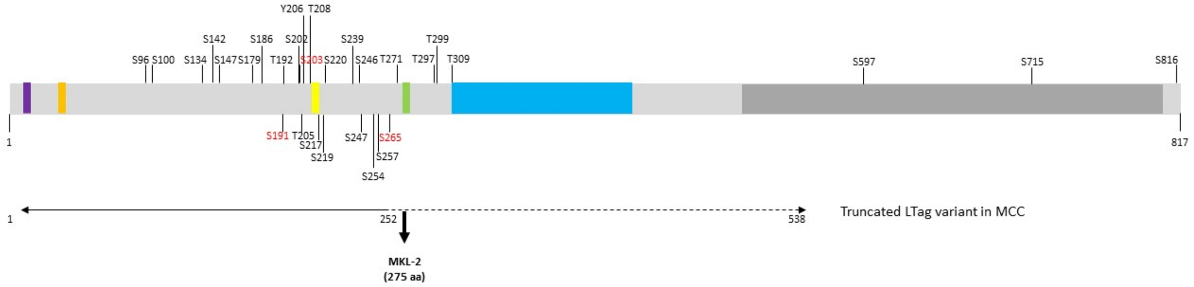

Figure S1. Proven and putative phosphorylation sites in MCPyV large T antigen. The functional domains of large T-antigen are shown in different colors: constant region 1 in purple (residues 13-17, LCKLL); DnaJ motif in orange (residues 42-48, HPDKGGN); retinoblastoma binding motif in yellow (residues 212-216, LFCDE); nuclear localization signal in green (residues 277-280, RKRK); origin binding domain in light blue (residues 308-433); helicase domain in dark grey). Truncated large T-antigen sequenced from Merkel cell carcinomas so far vary in length from 252 amino acids (JPN MCC74; BAO04662) to 538 amino acids (KIP1; AHN13646). A premature stop codon in MKL-2 indicated by the arrow results in a truncated large T-antigen that spans the N-terminal 275 residues.

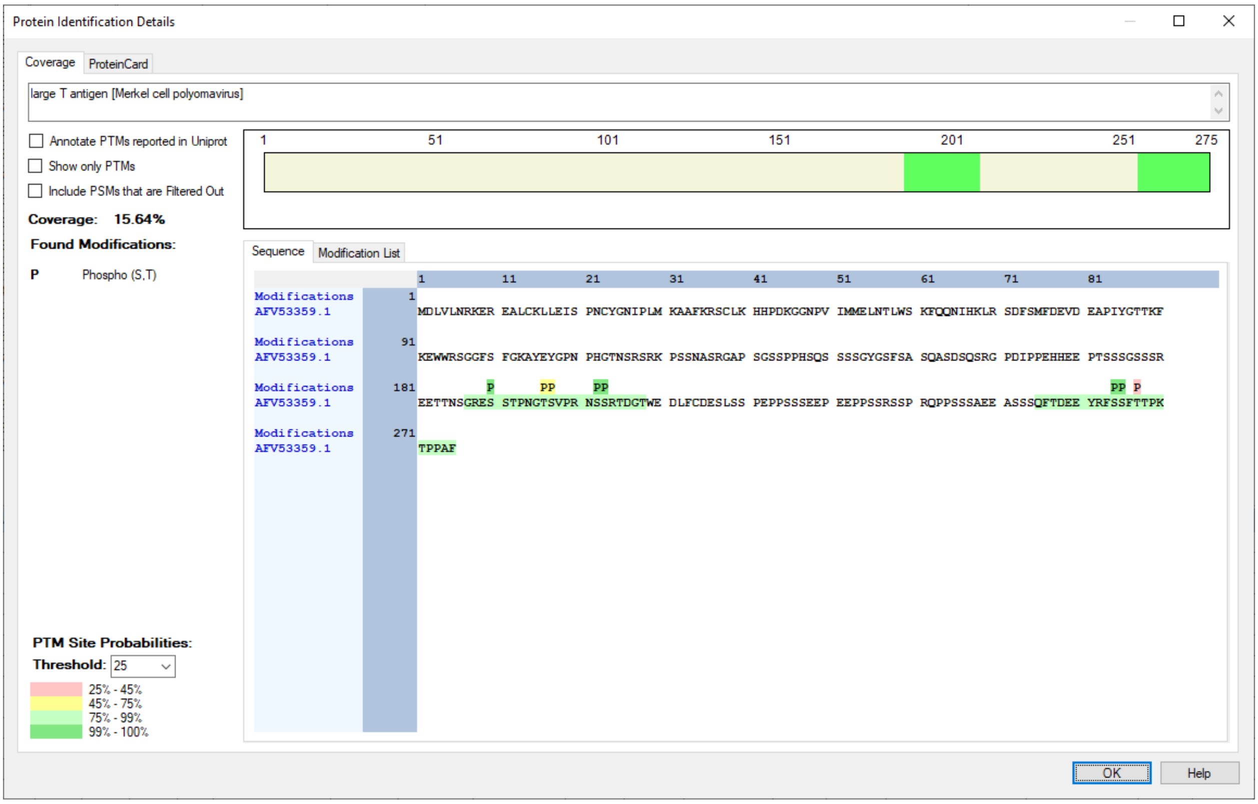

Figure S2. Phosphorylation sites detected by mass spectrometry of in vitro PKA phosphorylated peptides. The method does not allow distinction between phosphorylation of S202 and/or S203 (resp. S264 and/or S265).

A

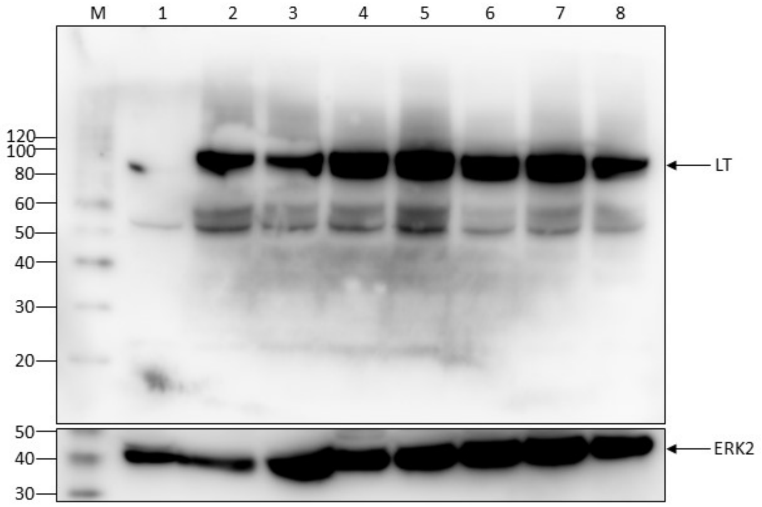

B

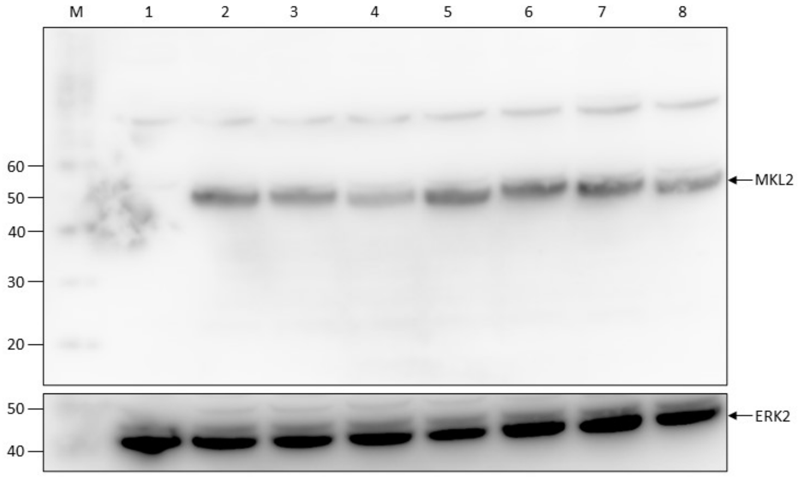

Figure S3. Expression levels of LTag, MKL2 LTag and their single mutants. (**A**) HEK293 cells were transfected with 1 mg empty expression vector (lane 1) or 1 mg of expression vector for respectively LT (lane 2), LT-S91A (lane 3), LT-S191D (lane 4), LT-S203A (lane 5), LT-S203D (lane 6), LT-S265A (lane 7) or LT-S265D (lane 8). (**B**) as A, but empty expression vector (lane 1), MKL2 (lane 2), MKL2-S91A (lane 3), MKL2-S191D (lane 4), MKL2-S203A (lane 5), MKL2-S203D (lane 6), MKL2-S265A (lane 7) or MKL2-S265D (lane 8). Lane M: protein marker (in kDa). ERK2 was used as a loading control.

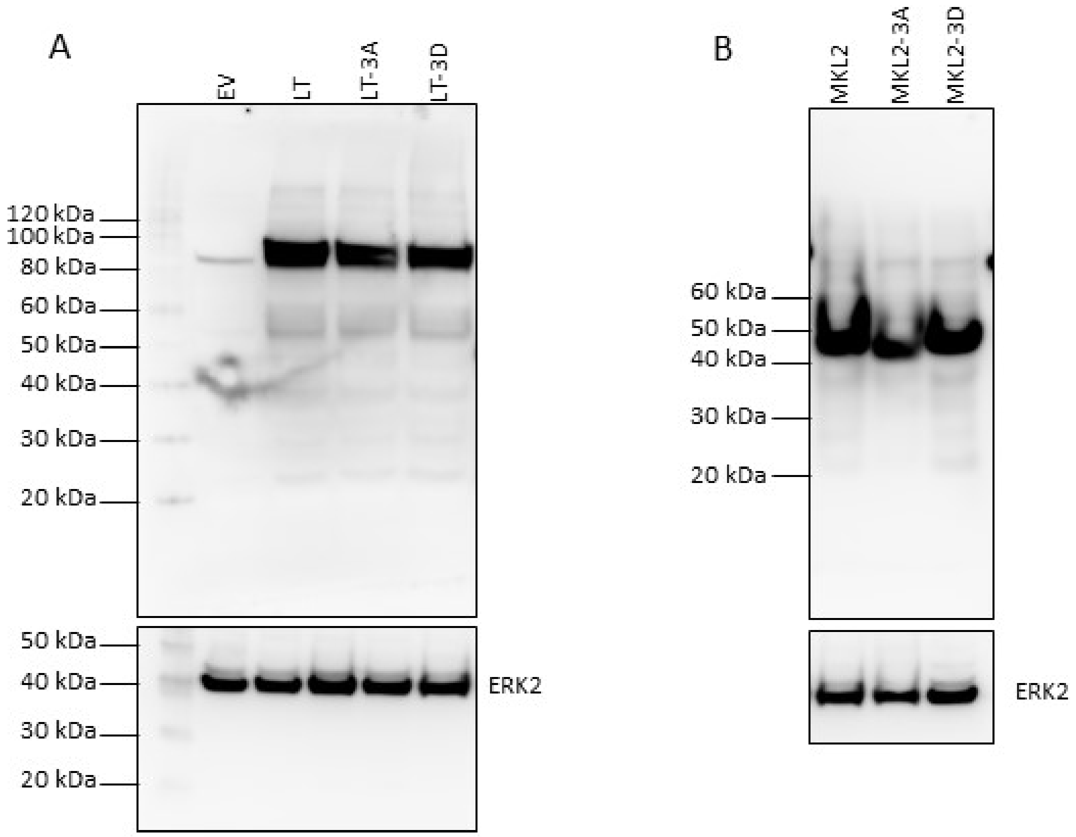

Figure S4. Expression levels of LTag, MKL2 LTag and their triple mutants. (**A**) HEK293 cells were transfected with empty expression vector (EV) or expression plasmids for MCPyV LTag, LT-3A, LT-3D and protein levels were determined by western blot. (**B**) as panel A but cells were transfected with expression plasmids for MKL2 tLT, MKL2-3A or MKL2-3D. ERK2 was used as a loading control. The protein size marker (in kDa) is shown.

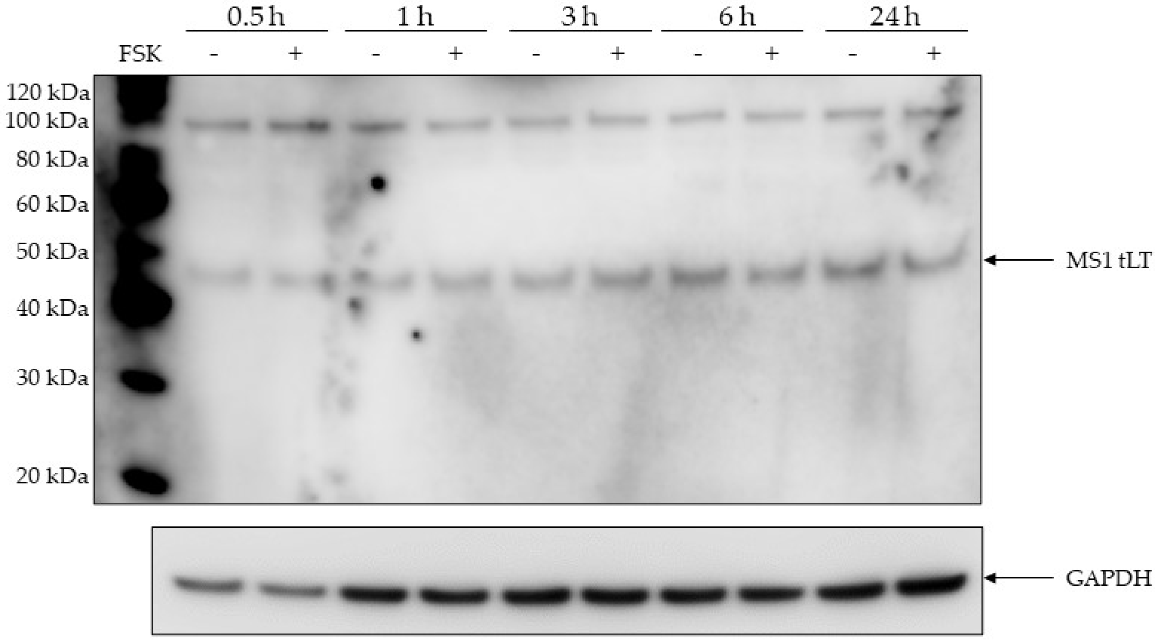

Figure S5. Activation of the PKA pathway does not increase the levels of MS1 LTag. Serum-starved MS1 cells were stimulated with 10 µM forskolin (FSK) for the indicated time points and levels of MS1 LT was monitored by western blotting using CM2B4 antibody. GAPDH was used as a loading control. The molecular marker (in kDa) is shown. The absence (-) or presence (+) of forskolin is indicated.

Table S1. Biological consequences of mutations in putative phosphoacceptor sites of MCPyV full-length (fl-LT) or truncated large T-antigen (tLT).

Mutation	fl-LT or tLT	Protein kinase	Effect	Reference
S96A	fl-LT	ND	no effect on half-life	[1]
S134A	fl-LT	ND	no effect on half-life	[1]
S142A	fl-LT	ND	increased half-life and reduced interaction with b-TrCP	[2]
S147A	fl-LT	ND	increased half-life; similar to wild-type LT, this mutant was unable to stimulate viral DNA replication; no effect on early and late promoter activity; reduced interaction with b-TrCP	[1] [2]
S179A	fl-LT	ND	no effect on half-life	[1]
S186A	fl-LT	ND	no effect on half-life	[1]
T192A/E [a]	tLT [b]	ND [c]	no effect on growth of MKL1 MCC cells; no effect on half-life	[3] [1]
S202A/E	tLT	ND	no effect on growth of MKL1 MCC cells	[3]
S203A/E	tLT	ND	no effect on growth of MKL1 MCC cells	[3]
T205A/E	tLT fl-LT	ND ND	no effect on growth of MKL1 MCC cells; T205A: no effect on hVam6p binding	[3] [4]
Y206A	fl-LT	ND	no effect on hVam6p binding	[4]
T208A	fl-LT	ND	no effect on hVam6p binding	[4]
S217A/E	tLT	ND	no effect on growth of MKL1 MCC cells	[3]
S219A/E	tLT	ND	S219A, but not S219E partially inhibited growth of MKL1 MCC cells [d]	[3]

S220A/E	tLT	ND	S220A, but not S220E inhibited growth of MKL1 MCC cells [e]; S220A impaired pRb binding and induction of E2F target genes; S220A: increase half-life and LT-dependent replication compared to wild-type LT; activation early and late promoter of an unmutated NCCR, but same activity as wild-type LT for a replication-deficient NCCR; S220A: reduced interaction with Skp2	[3] [1] [5] [2]
S239A/E	tLT	ND	no effect on growth of MKL1 MCC cells; S293A: increase half-life and LT-dependent replication compared to wild-type LT; activation early and late promoter of an unmutated NCCR, but same activity as wild-type LT for a replication-deficient NCCR; S239A: reduced interaction with Fbw7	[3] [1] [2]
S246A/S247A/S254A/T257A/S265A/T271A	tLT [f]	ND	no effect on nuclear import	[3]
S268A	fl-LT	ND	no effect on half-life	[1]
T271A	fl-LT	ND	no effect on ORI binding and replication of viral DNA; no effect on half-life	[6] [1]
T297A	fl-LT	ND	increased ORI binding and replication of viral DNA	[6]
T299A			reduced ORI binding and replication of viral DNA; no effect on half-life	[6] [1]
T309A	fl-LT	ND	no effect on half-life	[1]
S597A	fl-LT	ND	no effect on half-life	[1]
S715A	fl-LT	ND	no effect on half-life	[1]
S816A	fl-LT	ATM [g]	partially reverse inhibition of C33A cells growth compared to wild-type fl-LT; reduced apoptosis; no effect on half-life	[7] [1]

[a] Both nonphosphorylable A and phosphomimicking E mutants were tested; [b] N-terminal 278 amino acids; [c] not done; [d] 20% inhibition after 18 days; [e] 60% inhibition after 18 days; [f] N-terminal 334 amino acids; [g] ataxia-telangiectasia mutated.

Table S2. Results mass spectrometry of PKA-phosphorylated peptides.

Annotated Sequence in large T antigen [Merkel cell polyomavirus]	Modifications	Modification Pattern	# Isoforms	# Protein Groups	# Proteins	# PSMs	Master Protein Accessions	Positions in Master Proteins	Modifications in Master Proteins	# Missed Cleavages	Theo. MH+ [Da]	Confidence (by Search Engine): Sequest HT	XCorr (by Search Engine): Sequest HT	Sequence in Protein	Positions in Proteins
[T].DEEYRFSSFTTPKTPPAF.[-]		------------------	1	1	1	1	AFV53359.1	AFV53359.1 [258-275]		0	2119.99711	High	3.14	T.DEEYRFSSFTTPKTPPAF.-	[258-275]
[D].EEYRFSSFTTPKTPPAF.[-]		-----------------	1	1	1	5	AFV53359.1	AFV53359.1 [259-275]		0	2004.97017	High	4.33	D.EEYRFSSFTTPKTPPAF.-	[259-275]
[E].EYRFSSFTTPKTPPAF.[-]		----------------	1	1	1	6	AFV53359.1	AFV53359.1 [260-275]		0	1875.92758	High	3.72	E.EYRFSSFTTPKTPPAF.-	[260-275]
[E].EYRFSSFTTPKTPPAF.[-]	1xPhospho [S6(98.4)]	-----*----------	3	1	1	7	AFV53359.1	AFV53359.1 [260-275]	AFV53359.1 1xPhospho [S265(99)]	0	1955.89391	High	2.79	E.EYRFSSFTTPKTPPAF.-	[260-275]
[E].EYRFSSFTTPKTPPAF.[-]	1xPhospho [S5(98.7)]	----*-----------	3	1	1	8	AFV53359.1	AFV53359.1 [260-275]	AFV53359.1 1xPhospho [S264(99.5)]	0	1955.89391	High	4.05	E.EYRFSSFTTPKTPPAF.-	[260-275]
[E].EYRFSSFTTPKTPPAF.[-]	1xPhospho [S/T/Y]	positions not distinguishable	3	1	1	3	AFV53359.1	AFV53359.1 [260-275]		0	1955.89391	High	2.7	E.EYRFSSFTTPKTPPAF.-	[260-275]
[Q].FTDEEYRFSSFTTPKTPPAF.[-]		--------------------	1	1	1	5	AFV53359.1	AFV53359.1 [256-275]		0	2368.1132	High	4.71	Q.FTDEEYRFSSFTTPKTPPAF.-	[256-275]
[S].GRESSTPNGTSVPR.[N]		--------------	1	1	1	3	AFV53359.1	AFV53359.1 [187-200]		0	1444.71389	High	4.11	S.GRESSTPNGTSVPR.N	[187-200]
[S].GRESSTPNGTSVPRN.[S]		---------------	1	1	1	1	AFV53359.1	AFV53359.1 [187-201]		0	1558.75682	High	3.05	S.GRESSTPNGTSVPRN.S	[187-201]
[S].GRESSTPNGTSVPRN.[S]	1xPhospho [S4(99.4)]	---*-----------	1	1	1	2	AFV53359.1	AFV53359.1 [187-201]	AFV53359.1 1xPhospho [S190(99.4)]	0	1638.72315	High	3.07	S.GRESSTPNGTSVPRN.S	[187-201]
[S].GRESSTPNGTSVPRNS.[S]		----------------	1	1	1	3	AFV53359.1	AFV53359.1 [187-202]		0	1645.78885	High	3.53	S.GRESSTPNGTSVPRNS.S	[187-202]
[S].GRESSTPNGTSVPRNS.[S]	1xPhospho [S4(99.4)]	---*------------	1	1	1	2	AFV53359.1	AFV53359.1 [187-202]	AFV53359.1 1xPhospho [S190(99.4)]	0	1725.75518	High	2.71	S.GRESSTPNGTSVPRNS.S	[187-202]
[S].GRESSTPNGTSVPRNSS.[R]		-----------------	1	1	1	1	AFV53359.1	AFV53359.1 [187-203]		0	1732.82088	High	2.87	S.GRESSTPNGTSVPRNSS.R	[187-203]
[S].GRESSTPNGTSVPRNSSRTD.[G]		--------------------	1	1	1	6	AFV53359.1	AFV53359.1 [187-206]		0	2104.99661	High	4.56	S.GRESSTPNGTSVPRNSSRTD.G	[187-206]
[S].GRESSTPNGTSVPRNSSRTD.[G]	1xPhospho [S17(99.2)]	----------------*---	2	1	1	3	AFV53359.1	AFV53359.1 [187-206]	AFV53359.1 1xPhospho [S203(99.2)]	0	2184.96294	High	3.93	S.GRESSTPNGTSVPRNSSRTD.G	[187-206]
[S].GRESSTPNGTSVPRNSSRTD.[G]	1xPhospho [S16(99.4)]	---------------*----	2	1	1	2	AFV53359.1	AFV53359.1 [187-206]	AFV53359.1 1xPhospho [S202(99.4)]	0	2184.96294	High	4.38	S.GRESSTPNGTSVPRNSSRTD.G	[187-206]

Annotated Sequence in large T antigen [Merkel cell polyomavirus]	Modifications	Modification Pattern	# Isoforms	# Protein Groups	# Proteins	# PSMs	Master Protein Accessions	Positions in Master Proteins	Modifications in Master Proteins	# Missed Cleavages	Theo. MH+ [Da]	Confidence (by Search Engine): Sequest HT	XCorr (by Search Engine): Sequest HT	Sequence in Protein	Positions in Proteins
[S].GRESSTPNGTSVPRNSSRTDG.[T]		---------------------	1	1	1	2	AFV53359.1	AFV53359.1 [187-207]		0	2162.01807	High	3.55	S.GRESSTPNGTSVPRNSSRTDG.T	[187-207]
[S].GRESSTPNGTSVPRNSSRTDG.[T]	1xPhospho [T/S]	positions not distinguishable	2	1	1	3	AFV53359.1	AFV53359.1 [187-207]		0	2241.98441	High	2.66	S.GRESSTPNGTSVPRNSSRTDG.T	[187-207]
[S].GRESSTPNGTSVPRNSSRTDG.[T]	1xPhospho [S4(98.9)]	---*-----------------	2	1	1	1	AFV53359.1	AFV53359.1 [187-207]	AFV53359.1 1xPhospho [S190(98.9)]	0	2241.98441	High	2.68	S.GRESSTPNGTSVPRNSSRTDG.T	[187-207]
[S].GRESSTPNGTSVPRNSSRTDGT.[W]		----------------------	1	1	1	1	AFV53359.1	AFV53359.1 [187-208]		0	2263.06575	High	3.04	S.GRESSTPNGTSVPRNSSRTDGT.W	[187-208]
[S].QFTDEEYR.[F]		--------	1	1	1	1	AFV53359.1	AFV53359.1 [255-262]		0	1087.46908	High	1.97	S.QFTDEEYR.F	[255-262]
[S].QFTDEEYRF.[S]		---------	1	1	1	3	AFV53359.1	AFV53359.1 [255-263]		0	1234.53749	High	2.42	S.QFTDEEYRF.S	[255-263]
[S].QFTDEEYRFS.[S]		----------	1	1	1	3	AFV53359.1	AFV53359.1 [255-264]		0	1321.56952	High	2.53	S.QFTDEEYRFS.S	[255-264]
[S].QFTDEEYRFSS.[F]		-----------	1	1	1	2	AFV53359.1	AFV53359.1 [255-265]		0	1408.60155	High	2.63	S.QFTDEEYRFSS.F	[255-265]
[S].QFTDEEYRFSSF.[T]		------------	1	1	1	1	AFV53359.1	AFV53359.1 [255-266]		0	1555.66996	High	2.55	S.QFTDEEYRFSSF.T	[255-266]
[S].QFTDEEYRFSSFTTPKTPPAF.[-]		---------------------	1	1	1	13	AFV53359.1	AFV53359.1 [255-275]		0	2496.17178	High	4.13	S.QFTDEEYRFSSFTTPKTPPAF.-	[255-275]
[S].QFTDEEYRFSSFTTPKTPPAF.[-]	1xPhospho [S/T/Y]	positions not distinguishable	1	1	1	2	AFV53359.1	AFV53359.1 [255-275]		0	2576.13811	High	2.45	S.QFTDEEYRFSSFTTPKTPPAF.-	[255-275]
[S].SFTTPKTPPAF.[-]		-----------	1	1	1	1	AFV53359.1	AFV53359.1 [265-275]		0	1193.6201	High	2.91	S.SFTTPKTPPAF.-	[265-275]
[F].SSFTTPKTPPAF.[-]		------------	1	1	1	1	AFV53359.1	AFV53359.1 [264-275]		0	1280.65213	High	2.69	F.SSFTTPKTPPAF.-	[264-275]
[F].TDEEYRFSSFTTPKTPPAF.[-]		-------------------	1	1	1	2	AFV53359.1	AFV53359.1 [257-275]		0	2221.04479	High	3.66	F.TDEEYRFSSFTTPKTPPAF.-	[257-275]

Table S3. Primers used in this study.

name	Sequence (5′-3′)
S191A-Fw	CAGGAAGAGAATCCGCCACACCCAATGGAACC
S191A-Rv	GGTTCCATTGGGTGTGGCGGATTCTCTTCCTG
S191D-Fw	CAGGAAGAGAATCCGACACACCCAATGGAACC
S191D-Rv	GGTTCCATTGGGTGTGTCGGATTCTCTTCCTG
S203A-Fw	CCAGTGTACCTAGAAATTCTGCCAGAACGGATGGCACC
S203A-Rv	GGTGCCATCCGTTCTGGCAGAATTTCTAGGTACACTGG
S203D-Fw	CCAGTGTACCTAGAAATTCTGACAGAACGGATGGCACC
S203D-Rv	GGTGCCATCCGTTCTGTCAGAATTTCTAGGTACACTGG
S265A-Fw	GGAATACAGATTCTCCGCCTTCACCACCCCG
S265A-Rv	CGGGGTGGTGAAGGCGGAGAATCTGTATTCC
S265D-Fw	GGAATACAGATTCTCCGACTTCACCACCCCG
S265D-Rv	CGGGGTGGTGAAGTCGGAGAATCTGTATTCC
NCCR_GGAA_Fw	GCTAGGAGCCCCAAGAATCTGCCAACTTG
NCCR_GGAA_Rv	CAAGTTGGCAGATTCTTGGGGCTCCTAGC

References

1. Kwun, H.J.; Chang, Y; Moore, P.S. Protein-mediated viral latency is a novel mechanism for Merkel cell polyomavirus persistence. *Proc. Natl. Acad. Sci. USA* **2017**, *114*, E4040-e4047.
2. Nwogu, N.; Ortiz, L.E; Kwun,H.J. Merkel Cell Polyomavirus Large T Antigen Unique Domain Regulates Its Own Protein Stability and Cell Growth. *Viruses* **2020**, *12*, 1043.
3. Schrama, D.; Hesbacher, S.; Angermeyer, S.; Schlosser, A.; Haferkamp, S.; Aue, A.; Adam, C.; Weber, A.; Schmidt, M.; Houben, R. Serine 220 phosphorylation of the Merkel cell polyomavirus large T antigen crucially supports growth of Merkel cell carcinoma cells. *Int. J. Cancer* **2016**, *138*, 1153-62.
4. Liu, X.; Hein J.; Richardson, S.C.; Basse, P.H.; Toptan, T.; Moore, P.S.; Gjoerup, O.V.; Chang, Y. Merkel cell polyomavirus large T antigen disrupts lysosome clustering by translocating human Vam6p from the cytoplasm to the nucleus. *J. Biol. Chem.* **2011**, *286*, 17079-90.
5. Alvarez Orellana, J.; Kwun, H.J.; Artusi, S.; Chang, Y.; Moore, P.S. Sirolimus and Other Mechanistic Target of Rapamycin Inhibitors Directly Activate Latent Pathogenic Human Polyomavirus Replication. *J. Infect. Dis.* **2021**, *224*, 1160-1169.
6. Diaz, J.; Wang, X.; Tsang, S.H.; Jiao, J.; You, J. Phosphorylation of large T antigen regulates Merkel cell polyomavirus replication. *Cancers (Basel)* **2014**, *6*, 1464-86.
7. Li, J.; Diaz, J.; Wang,X.; Tsang, S.H.; You, J. Phosphorylation of Merkel cell polyomavirus large tumor antigen at serine 816 by ATM kinase induces apoptosis in host cells. *J. Biol. Chem.* **2015**, *290*, 1874-84.
